# High-Temperature Oxidation and Wear Resistance of TiAlSiN/AlCrN Multilayer Coatings Prepared by Multi-Arc Ion Plating

**DOI:** 10.3390/nano15070503

**Published:** 2025-03-27

**Authors:** Jie Liu, Haijuan Mei, Junfang Hua, Juan Wang, Yongchao Wang, Genmiao Yi, Xin Deng

**Affiliations:** 1 School of Intelligent Manufacturing, Guangzhou Panyu Polytechnic, Guangzhou 511483, China; liujie@gzpyp.edu.cn (J.L.); huajf@gzpyp.edu.cn (J.H.); wangyongc@gzpyp.edu.cn (Y.W.); yigm@gzpyp.edu.cn (G.Y.); 2 Guangdong Provincial Key Laboratory of Electronic Functional Materials and Devices, Huizhou University, Huizhou 516007, China; 3 School of Art and Design, Guangdong University of Technology, Guangzhou 510006, China; wangjuan@gdut.edu.cn; 4 School of Electromechanical Engineering, Guangdong University of Technology, Guangzhou 510006, China

**Keywords:** multilayer, TiAlSiN, AlCrN, oxidation resistance, wear, mechanical properties

## Abstract

TiAlSiN and AlCrN coatings are two representative coatings with excellent properties in TiN-based and CrN-based coatings, respectively. Multilayering is one of the most important directions for coating performance optimization. In this paper, nanoscale monolayer TiAlSiN, AlCrN, and multilayer TiAlSiN/AlCrN coatings were prepared. The microstructure, mechanical properties, oxidation resistance, and wear resistance of the above three coatings were investigated. The following properties of the TiAlSiN/AlCrN coating, including phase, nanohardness, elastic modulus, adhesion strength, and oxidation resistance, fall between those of the TiAlSiN and AlCrN coatings and conform to the “law of mixtures”. Due to the interfacial effect of the multilayer coating, the residual stress of the TiAlSiN/AlCrN coating is less than that of the two monolayer coatings. At 500 °C, the order of wear resistance of the three coatings is consistent with the order of H^3^/E*^2^ values, i.e., TiAlSiN > TiAlSiN/AlCrN > AlCrN; at 800 °C, the order of wear resistance becomes TiAlSiN/AlCrN > TiAlSiN > AlCrN because TiAlSiN coating has entered the rapid oxidization stage first, reducing its wear resistance.

## 1. Introduction

Among Ti-based and Cr-based coatings, TiN and CrN are the two earliest and most widely used. Their performance can be further improved by adding other elements. Al and Si are the two most successful additive elements. Al atoms strengthen solid solutions by replacing Ti in the lattice of TiN, but too much Al will transform TiAlN from a single FCC phase to a mixed phase structure of FCC and HCP, decreasing the coating’s mechanical properties. In addition, the dense and chemically stable Al_2_O_3_ generated by the oxidation of Al is also responsible for the improved resistance to oxidation of Al-containing coatings. AlTiN- and AlCrN-coated tools have been successfully used in machining because of their excellent hardness and high-temperature oxidation resistance [1,2]. Since TiO_2_ undergoes a phase transition from dense α-TiO_2_ to loose r-TiO_2_ at high temperatures, whereas Cr_2_O_3_ does not have such a change, AlCrN has superior resistance to oxidation than AlTiN.

Si was added to TiAlN to obtain TiAlSiN coating. Nanocomposite structures have been proven to exist in such coating [3,4]. This structure consists of amorphous Si_3_N_4_ surrounding TiN grains, while Al is solidly dissolved in the TiN lattice. This nanocomposite structure hinders dislocations and refines the grains, thus enhancing the coating’s hardness [5], which will contribute to its wear resistance. Coating wear resistance is also related to the modulus of elasticity E. Many researchers have used the H/E* and H^3^/E*^2^ values to comprehensively evaluate the effects of hardness and modulus of elasticity on coating wear resistance [6,7]. The nanocomposite structure can inhibit the TiN grains encapsulated by the amorphous phase from growing up at high temperatures and also prevent the intrusion of oxygen, so TiAlSiN obtains excellent high-temperature stability, as well as oxidation resistance [8,9]. This will help it be used in high-temperature, high-stress applications such as high-speed machining of difficult-to-cut materials [10,11].

In recent years, making coatings into multilayers has become an effective means of obtaining comprehensive coating performance. Due to their unique interfacial effects, multilayer coatings offer outstanding toughness, corrosion resistance, and thermal stability over monolayer coatings [12]. In the study of CrN/ZrN [13] multilayer coatings, the oxidation resistance of the coatings was found to increase as the number of layers increased. The modulation period has a decisive influence on multilayer coatings’ microstructure and mechanical properties. In the study of TiAlSiN/CrN [14] multilayer coatings, it was found that the columnar crystal grain size increased with decreasing modulation period, while the hardness and elastic modulus first increased and then decreased. Adjusting the composition and thickness of the different layers within multilayer coatings can improve the coating’s wear resistance. In the study of AlCrBN/AlTiBN [15] multilayer coatings, the lowest wear rate of 8.7 ± 2.3 × 10^−7^ mm^3^/Nm was obtained when the thicknesses of AlTiBN and AlCrBN were set to 6.5 nm and 16.7 nm, respectively. Baijun Xiao [16], in his experiments on turning SKD11 (equivalent to AISI D2 steel), found that the AlCrN/AlTiSiN-coated tool had a longer life of about 800 m compared to the monolayer AlCrN and AlTiSiN coatings. This can be attributed to its higher hardness, adhesion strength, and resistance to oxidation.

Past studies have focused on the properties or preparation of TiAlSiN or AlCrN coatings. However, fewer comparative studies have been conducted between the two coatings and the multilayer coating made from both of them, and the conclusions have not been inconsistent.

## 2. Materials and Methods

### 2.1. Coating Deposition

**Figure 1:**
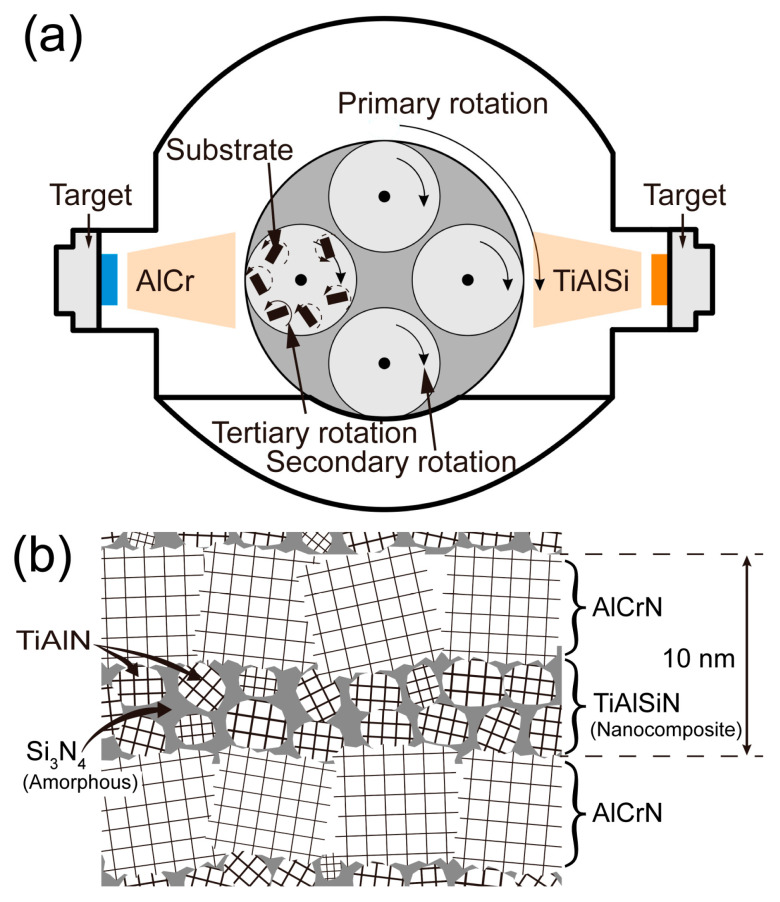
Illustration of coating fabrication and coating structure: (**a**) fabrication equipment for multilayer coating; (**b**) structure of multilayer coating.

The coatings in this paper were manufactured using the multi-arc ion plating technique (NH-10758 multifunctional PVD coater, Dongguan Nahu Crystal Materials Co., Dongguan, China). The coated substrate was fitted on a rotating shelf inside the furnace chamber and has three dimensions of rotational motion, as illustrated in Figure 1a. For monolayer coating, targets are loaded on one side only, either on the left or right; for multilayer coating, targets of different compositions are loaded on opposite sides. The structure and composition of TiAlSiN/AlCrN multilayer coating were designed according to Figure 1b. The modulation period of the multilayer coating is determined by the rotational speed of the rotating shelf. In our team’s published study [17], the rotational speed of the rotating shelf was set to 2 r/min, and TiAlSiN/AlCrN multilayer coating with a 20 nm modulation period was obtained. This nanoscale modulation period was confirmed by TEM. In this paper, the rotational speed of the rotating shelf was doubled to 4 r/min, and a 10 nm modulation period of TiAlSiN/AlCrN multilayer coating will be obtained.

The coated substrates are as follows: a WC cemented carbide block with a Co mass fraction of 6% and a size of 18 × 18 × 5 mm for mechanical property testing and fracture cross-section observation; single-crystal alumina with an outer diameter of 51 mm and a thickness of 0.5 mm for high-temperature oxidation test; a polycrystalline alumina with dimensions of 23 × 10 × 0.5 mm for thermogravimetric analysis (TGA); and an AISI 304 stainless steel sheet with dimensions of 60 × 10 × 0.8 mm for residual stress testing. The substrates were polished, cleaned, and put into the furnace chamber, which was evacuated to 5.0 × 10^−3^ Pa and heated to 500 °C. Next, the substrate was glow-cleaned for 9.5 min by passing 0.6 Pa argon gas at a bias voltage of 800 V and was then anodically etched for 20 min at a bias voltage of 300 V to further clean and activate the substrate surface. Then, nitrogen was passed in three stages at pressures of 2 Pa, 3 Pa, and 3 Pa, corresponding to bias voltages of 30 V, 40 V, and 60 V, and target currents of 145 A, 160 A, and 160 A, respectively. The above changes form a gradient layer, reducing the residual stress and enhancing the adhesion between the coating and the substrate. To achieve the same thickness of 3.5 μm for all three coatings, the deposition times of TiAlSiN, AlCrN, and TiAlSiN/AlCrN coatings were 194 min, 111 min, and 74 min, respectively. Finally, the samples were taken out after the temperature was lowered to room temperature.

### 2.2. Coating Characterization

The surface and fracture cross-section morphology and composition of the coatings were measured using a Nova NanoSEM 430 (FEI, Eindhoven, The Netherland). Phase analysis of the coatings was carried out using a Bruker D8 Advance X-Ray Diffractometer (Bruker Corp, Billerica, MA, USA). The parameters were set to a 2θ angle of 25°~85°, a step size of 0.02°, a dwell time of 0.5 s per step, and an incidence angle of 1° for grazing incidence XRD (GIXRD). The dwell time per step for conventional X-ray diffraction (XRD) was adjusted to 0.3 s. The coatings’ nanohardness (H) and elastic modulus (E) were measured by nanoindentation using Anton Paar’s TTX-NHT2 (Anton Paar, Graz, Austria). Test conditions were established with an applied load of 10 mN, a loading rate of 15 mN/min, a maximum depth of 120 nm, and a load retention time of 5 s at the maximum depth. It was necessary to restrict the indentation depth to 10% of the coating thickness in order to minimize substrate interference with the coating’s nanohardness. The adhesion strength between the coating and the substrate was measured using an Anton Paar RST3 Scratch-meter (Anton Paar, Graz, Austria). The parameters were set as a diamond tip diameter of 200 μm, an applied load ranging from 1 to 100 N with a loading rate of 200 N/min, and a scratch length of 3 mm. The residual stresses in the coating were measured using the Supro Instruments FST-1000 Film Stress Tester (SuPro Instruments Ltd., Shengzhen, China) and then calculated using the Stoney equation [18]. TGA testing of the coatings was carried out using a NETZSCH STA449F5 thermogravimetric analyzer (NETZSCH Group, Bavarian, Germany). The heating rate was 10 K/min, and the dry airflow was maintained at 50 sccm during the entire experiment. Oxidation experiments on the coatings were carried out in a TSX1700 muffle furnace (Cinite, Beijing, China). The coatings were exposed to temperatures of 800 °C, 950 °C, and 1050 °C with a 3 h dwell time.

The coatings’ resistance to wear was tested using an Anton Paar THT high-temperature ball and disk friction tester (Anton Paar, Graz, Austria). The counterbody was a polycrystalline Al₂O₃ ball, measuring 6 mm in diameter. The test parameters were load 10 N, linear velocity 0.1 m/s, friction radius 2 mm, and the number of friction circles 8000 at 500 °C and 6000 at 800 °C, equivalent to a distance of 100 m and 75 m, respectively. A lower number of cycles is set at higher temperatures to prevent the coating from being worn through at elevated temperatures.

The formula for calculating the coating wear rate is as follows:

(1) $$W=\frac{V}{L\times P}$$

where *W* is the wear rate, *V* is the volume of material lost in the friction experiment in mm^3^, *L* is the total length of the friction in m, and *P* is the load in N. *V* is equal to the product of the cross-sectional area of the wear track and the circumference of the circle, the cross-sectional area of the wear track is measured in the laser confocal software, and L is calculated as the product of the number of circles and the circumference.

Surface roughness tends to make the nanoindentation data discrete. Five randomly selected test points on the surface were needed, and the data obtained were evaluated for variability by calculating the mean and standard deviation. Outliers were identified using the Grubbs test and excluded if they were significantly out of the expected range. Scratch, residual stress, and cross-sectional area of the wear track were measured three times each to ensure data reliability and repeatability.

## 3. Results and Discussion

### 3.1. Phase and Microstructure

**Figure 2:**
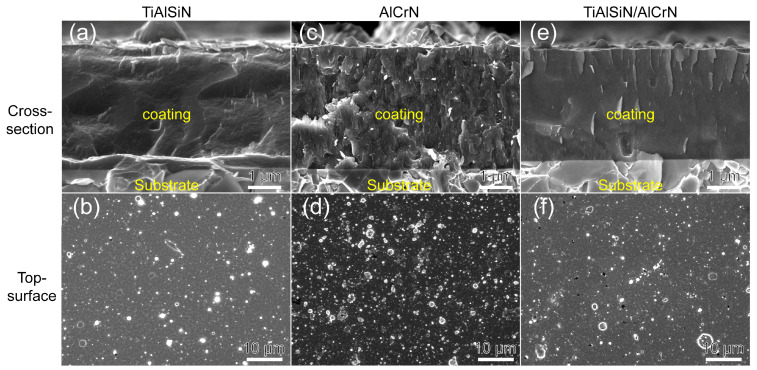
Morphology of fracture cross-section and top surface for three coatings: (**a**,**b**) TiAlSiN; (**c**,**d**) AlCrN; (**e**,**f**) TiAlSiN/AlCrN.

Figure 2 presents the fracture cross-sections and surface morphologies of the TiAlSiN, AlCrN, and TiAlSiN/AlCrN coatings. The AlCrN coating exhibits distinct columnar crystal structures, whereas the TiAlSiN and TiAlSiN/AlCrN coatings display featureless morphologies, typical of Si-containing coatings. Numerous white microparticles are evenly distributed across the surface of the coatings, which exhibits a typical deposition pattern of arc ion plating. This is because the cathodic arc produces an arc spot on the target’s surface with a small breakdown area and, thus, a high-power density. Under the combined effect of internal expansion pressure and negative bias pressure at the substrate end, the metal in the molten pool splashes onto the substrate surface and forms particles before it can evaporate [19,20]. Among these target materials, Al has the lowest melting point. Additionally, the AlCrN coating contains the highest Al content among the three coatings. Consequently, the AlCrN coating exhibits the most white microparticles on its surface, followed by the TiAlSiN/AlCrN coating.

The chemical compositions of the three coatings were analyzed using EDS, and the results are presented in Table 1. The elemental ratios closely match the target. However, the Si content is lower than the target due to its lower deposition rate compared to Al and Ti.

**Figure 3:**
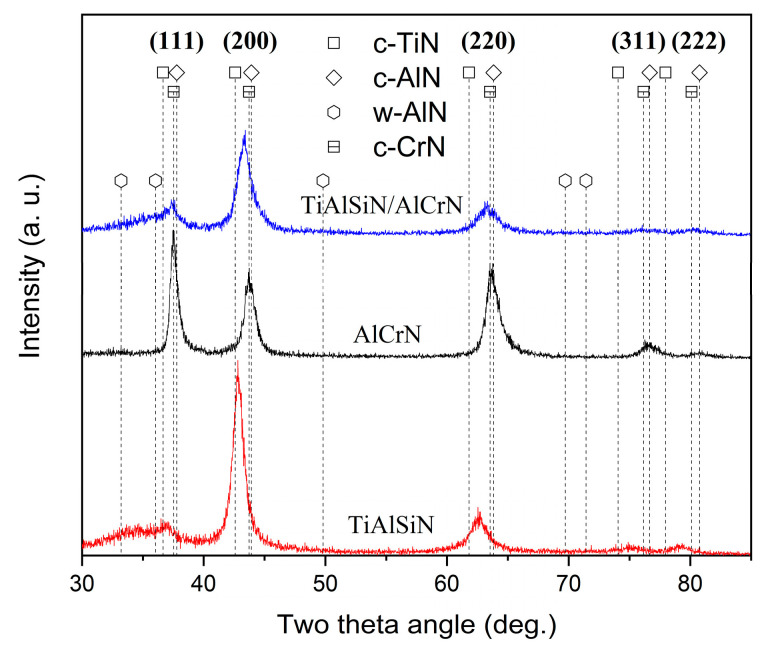
XRD patterns of three coatings.

Figure 3 shows the phase analysis results of the coatings obtained using GIXRD. According to ICDD No. 38-1420 for c-TiN and ICDD No. 11-0065 for c-CrN, the diffraction peaks corresponding to the (111), (200), (220), (311), and (222) planes of the cubic phases of c-TiAlN and c-CrAlN are observed in both TiAlSiN and AlCrN coatings. The TiAlSiN coating exhibits a pronounced TiN (200) plane preferred orientation, whereas the AlCrN has a CrN (111) preferred orientation. The diffraction peaks of TiAlSiN/AlCrN coating appear between the standard peaks of c-TiN and c-CrN, indicating that the TiAlSiN/AlCrN coating exhibits a mixed cubic phase structure of c-TiAlCrN, characterized by a preferred orientation along the (200) plane. Distinct w-AlN phases (ICDD No. 25-1133) are observed in both the TiAlSiN and TiAlSiN/AlCrN coatings but not in the AlCrN coating, despite the higher Al content (70 at.%) present in the AlCrN coating. Other researchers have made similar findings [21]. This is attributed to the fact that the solubility of Al in TiN is less than that of CrN, and the introduction of Si further reduces the solubility of Al in TiN. Both factors result in the formation of w-AlN phases in the TiAlSiN and TiAlSiN/AlCrN coatings.

### 3.2. Mechanical Properties

**Figure 4:**
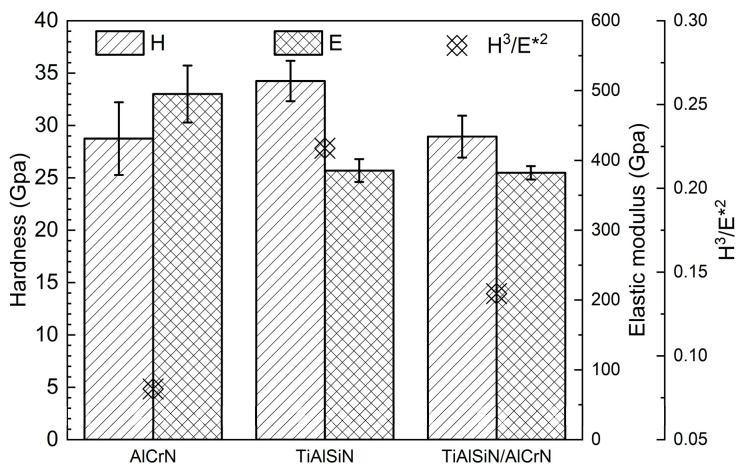
Nanohardness and elastic modulus of three coatings.

The nanohardness and elastic modulus of TiAlSiN, AlCrN, and TiAlSiN/AlCrN coatings are presented in Figure 4. The highest nanohardness and lowest elastic modulus are obtained for the TiAlSiN coating, while these two metrics for the TiAlSiN/AlCrN coating are in between those for the TiAlSiN and AlCrN monolayer coatings, which is by the “law of mixtures”. A coating’s plastic deformation and wear resistance can also be usually measured by H^3^/E*^2^ [22,23], where E* is equal to E(1 − μ^2^). The higher the value of H^3^/E*^2^, the higher the coating’s plastic deformation and wear resistance. Since TiAlSiN coating has the highest nanohardness and lowest modulus of elasticity, it has the highest H^3^/E*^2^ value.

**Figure 5:**
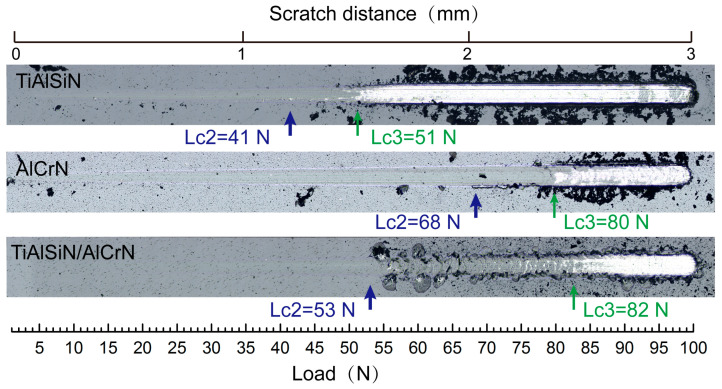
The critical Lc2 value and corresponding optical graphs after the scratch test of three coatings.

The adhesion strength between the coating and the substrate is one of the most important factors in evaluating a coating’s performance. Figure 5 presents the critical Lc2 and Lc3 values of the three coatings measured during the scratch test. Lc2 represents the load at which cracks initiate and minor coating flaking occurs, whereas Lc3 indicates the load at which the substrate becomes exposed. Typically, Lc2 is regarded as the critical load for coating failure and serves as a measure of coating adhesion strength. The AlCrN coating exhibits the highest adhesion strength. In comparison, the TiAlSiN coating exhibits an Lc2 value of only 41 N, whereas the adhesion strength of the TiAlSiN/AlCrN coating falls between the other two coatings. The significant difference between the Lc2 and Lc3 values for the multilayer coating, along with the presence of powdery debris distributed on both sides of the scratches, indicates that the multilayer coating tends to gradually detach from the substrate in thin layers. Conversely, the slight difference between the Lc2 and Lc3 values of the two monolayer coatings indicates that these coatings are completely removed quickly upon reaching the critical load (Lc2). This rapid detachment is confirmed by the presence of large debris fragments on both sides of the scratches. After the addition of Si, the poor bonding of amorphous Si₃N₄ to the substrate crystals and the higher residual stresses within the coating result in reduced adhesion strength of the TiAlSiN coating [23]. The residual stress values of the three coatings are −8.3 GPa for TiAlSiN, −5.8 GPa for AlCrN, and −3.6 GPa for TiAlSiN/AlCrN, respectively. The lower stresses in the multilayer coating are attributed to the interfacial effect of the increased number of coating interfaces, which effectively release the internal stresses throughout the coating.

### 3.3. High-Temperature Oxidation Resistance

**Figure 6:**
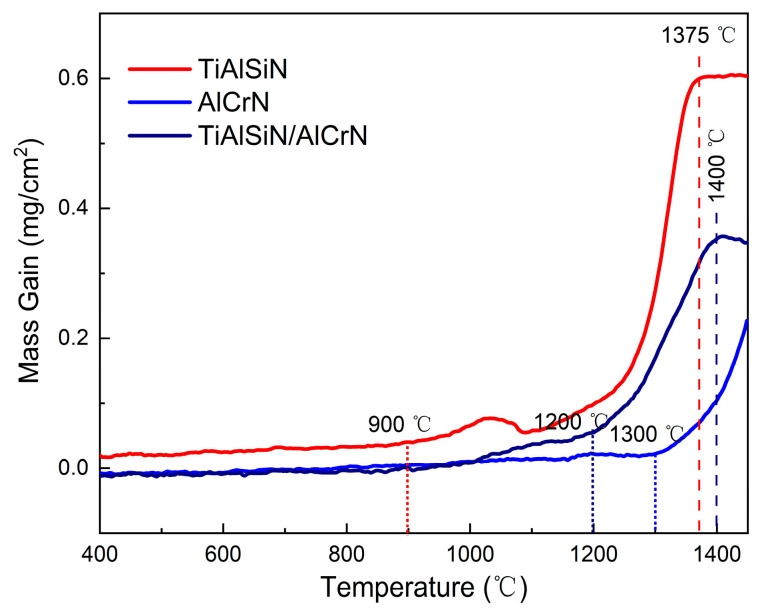
TGA curves of three coatings in synthetic air atmosphere.

Figure 6 depicts the TGA curves of TiAlSiN, AlCrN, and TiAlSiN/AlCrN coatings subjected to continuous heating from room temperature to 1450 °C in synthetic air. The oxidized weight gain of the three coatings is imperceptible until 900 °C. From 950 °C to 1050 °C, the TiAlSiN coating’s TGA curve suddenly forms a local peak upward, indicating the emergence of some kind of new oxidation mechanism. Further, the TiAlSiN coating enters a rapid oxidation stage. As the temperature increases to 1375 °C, the oxidation weight gain of the TiAlSiN coating remains unchanged, signifying that the coating has undergone complete oxidation and reached the terminated oxidation stage. On the contrary, the oxidation of the AlCrN coating is not complete by the end of the experiment at 1450 °C, suggesting that the oxidation resistance of the AlCrN coating exceeds that of TiAlSiN. Meanwhile, the initial and termination oxidation temperatures of the TiAlSiN/AlCrN multilayer coating are 1200 °C and 1400 °C, respectively, between the two monolayer coatings.

**Figure 7:**
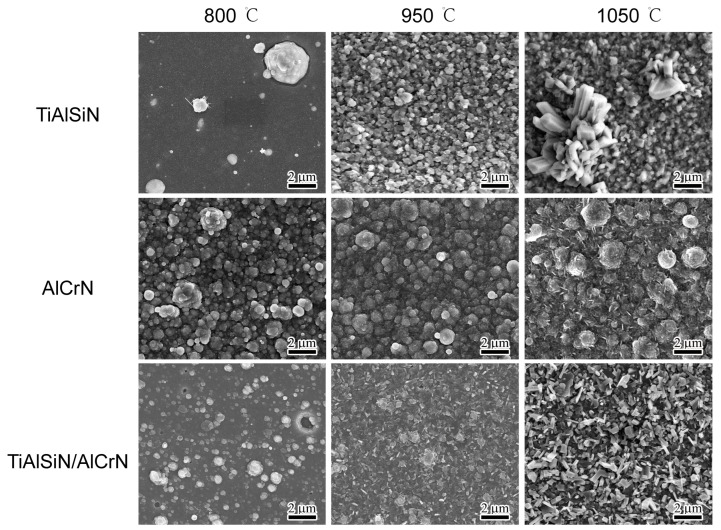
Morphology of top surface for three coatings after oxidizing at high temperature for three hours.

For the oxidation details of the coatings, the three coatings were oxidized at 800 °C, 950 °C, and 1050 °C for three hours. Figure 7 shows the surface morphology after oxidation. From 800 °C to 1050 °C, the surface of the TiAlSiN coating undergoes the greatest change, with the number of oxides increasing significantly and the oxide particles gradually becoming larger. While the surface of the AlCrN coating has the smallest change, the magnitude of change in the TiAlSiN/AlCrN coating is between the above two.

**Figure 8:**
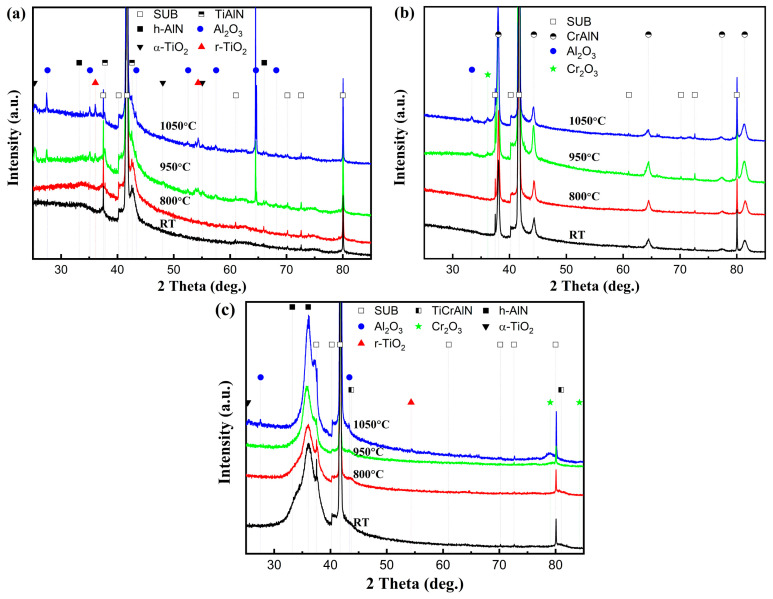
XRD patterns of coatings after high-temperature oxidation tests: (**a**) TiAlSiN; (**b**) AlCrN; (**c**) TiAlSiN/AlCrN.

XRD was performed on the oxidized surfaces to identify the oxidation products, and the results are presented in Figure 8. At 800 °C, no oxide peaks appear on the surface of any of the three coatings. At 950 °C, three oxides, Al_2_O_3_ (ICDD No. 10-0173, ICDD No.26-0031, ICDD No. 46-1131), α-TiO_2_ (ICDD No. 21-1272), and r-TiO_2_ (ICDD No. 21-1276), appeared in the TiAlSiN coating, as shown in Figure 8a. At 1050 °C, the peak intensity of Al_2_O_3_ was enhanced, especially at a diffraction peak of 64.5°. The peak intensities of the two oxides of TiO_2_ show opposite trends, with α-TiO_2_ weakening, e.g., 25.2°, 48.0°, and 55.0°, and r-TiO_2_ enhancing, e.g., 36.0° and 54.3°, suggesting that TiO_2_ is in the process of phase transition from α-TiO_2_ to r-TiO_2_ from 950 °C to 1050 °C. The onset temperature of this phase transition is after 800 °C and before 950 °C. It is consistent with the findings of Kumar [24] and Shi [25]. They suggested that the phase transition from α-TiO_2_ to r-TiO_2_ occurs gradually between 800 °C and 1000 °C and is completed at 1100 °C to 1200 °C. The phase transition of TiO_2_ explains the local peak near 1050 °C in the TGA curve of the TiAlSiN coating in Figure 6. From 950 °C to 1050 °C, the enhancement of the oxide peaks of both AlCrN and TiAlSiN/AlCrN coatings is almost negligible. The weaker intensity of their oxide peaks compared with TiAlSiN indicates that their oxidation resistance is significantly superior to that of TiAlSiN. The above results are consistent with the TGA results in Figure 6.

**Figure 9:**
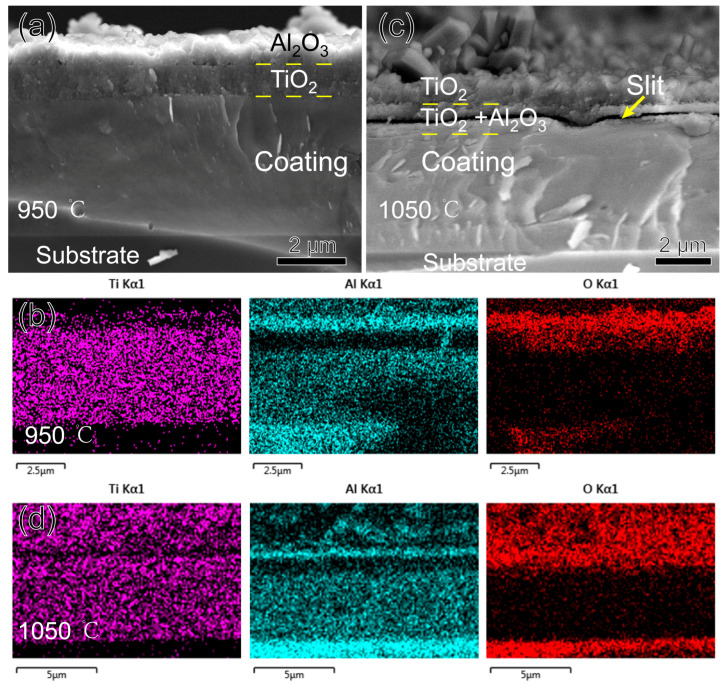
Morphology and EDS mapping of TiAlSiN coating fracture cross-section after oxidizing at high temperature for three hours: (**a**,**b**) 950 °C; (**c**,**d**) 1050 °C.

SEM observations and EDS mapping were conducted on the fracture cross-sections of the three coatings oxidized at 950 °C and 1050 °C, respectively. At 950 °C, the oxides of the TiAlSiN coating showed a bilayer structure, as shown in Figure 9a,b. With reference to the XRD results, the upper layer is Al_2_O_3_, and the lower layer is TiO_2_. Since ∆G^0^ = −1336 KJ/mol for Al_2_O_3_ and ∆G^0^ = −753 KJ/mol for TiO_2_, Al_2_O_3_ is preferentially generated and located in the upper layer. The dense Al_2_O_3_ film covering the surface of the coating can block the diffusion of oxygen into the interior of the coating, thus improving the high-temperature oxidation resistance of the coating [26]. Since the thermal expansion coefficient of TiO_2_ (10.5 × 10^−6^/K) is larger than that of Al_2_O_3_ (8.4 × 10^−6^/K) [27], when the temperature continues to increase to 1050 °C, the upper layer of Al_2_O_3_ is tensile cracked. The unoxidized and highly concentrated Ti in the lower layer diffuses along the crack to the upper layer. Therefore, as seen in Figure 9d, the upper layer of the oxide is transformed into a Ti-rich oxide, and the lower layer is a mixed Al-Ti oxide.

Compared to α-TiO_2_, r-TiO_2_ is loose and porous, so when it appears in large quantities, it is accompanied by the appearance of a large number of pores within the oxide, which allows for an additional mode of diffusion of oxygen and metal by mass transport in addition to the original short-circuit diffusion. The whole oxide layer is filled with r-TiO_2_ from phase transition at 1050 °C, providing a convenient channel for the outward migration of Ti. When a significant amount of Ti diffuses away from the oxide–nitride interface, micropores are formed, and the micropores are further connected to form a slit, as shown in Figure 9c. Even so, oxidation at 1050 °C for three hours is slow, and the coating’s oxidized thickness is less than 15% of its total thickness. The outstanding oxidation resistance of the TiAlSiN coating is attributed to the presence of Si. First, the nanocomposite structure of the coating extends the oxygen diffusion path; second, Si has the effect of preventing the transformation of TiO_2_ from dense α-TiO_2_ to looser r-TiO_2_, which retards the oxidation of the coating [28]; and third, SiO_2_, an oxide of Si, also plays a protective role in the antioxidant property of the coating [29]. SiO_2_ yields were very low and not detected in the EDS or XRD test.

**Figure 10:**
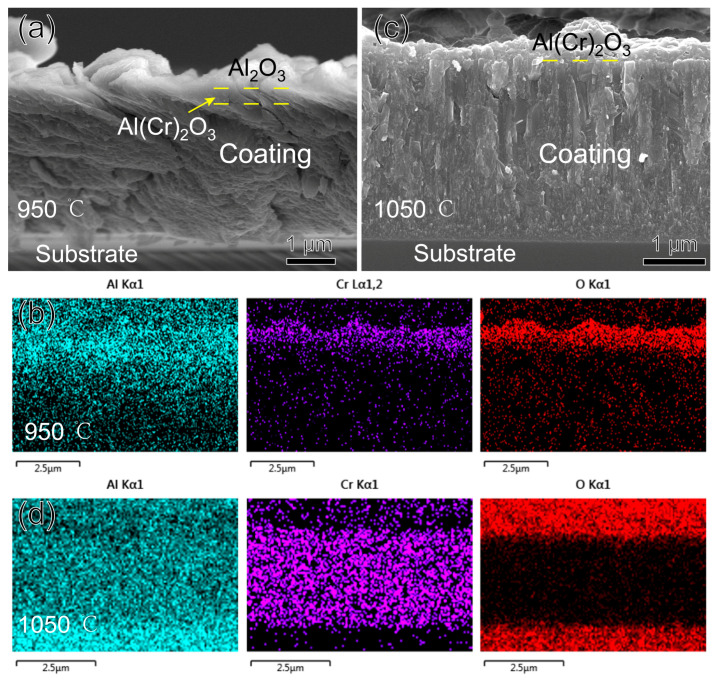
Morphology and EDS mapping of AlCrN coating fracture cross-section after oxidizing at high temperature for three hours: (**a**,**b**) 950 °C; (**c**,**d**) 1050 °C.

Figure 10 shows the morphology and EDS mapping of the fracture cross-section of the AlCrN coating after oxidation for three hours at 950 °C and 1050 °C. The oxide layer of the coating at 950 °C is also double-layered, as shown in Figure 10a,b. From the corresponding Al_2_O_3_ and Cr_2_O_3_ at 33.3° and 36.2°, respectively, in Figure 8b, it can be seen that the upper layer of the oxide is Al_2_O_3_, and the lower layer is a mixture of Al_2_O_3_ and Cr_2_O_3_, as observed by Xiang D. Zhang [30]. Al_2_O_3_ is located in the upper layer for the same reason because its ∆G^0^ is much lower than that of Cr_2_O_3_, which is −845 KJ/mol, so Al_2_O_3_ is preferentially generated and located in the upper layer. At 1050 °C, the delamination at 950 °C is no longer present, and the whole oxide layer is a mixture of Al_2_O_3_ and Cr_2_O_3_. AlCrN coating did not replace the upper oxide layer at 1050°C like TiAlSiN coating because the thermal expansion coefficient of Cr_2_O_3_ is only 7 × 10^−6^/K, less than that of Al_2_O_3_, and the thermal stresses could not tear apart the upper Al_2_O_3_ and release the Cr ions. However, at this temperature, the diffusion rate of Cr ions inside the oxide dramatically increases compared to Al [31,32]. The Cr gathered in the lower layer diffuses upwards and mixes with Al. We already know that Al_2_O_3_ and Cr_2_O_3_ have the same hexagonally symmetric corundum crystal structure, and the final formation of Cr_2_O_3_ and Al_2_O_3_ exists as a solid solution of (Cr, Al)_2_O_3_. Assuming a higher temperature or a longer duration of the experiment, delamination of the oxide with Cr_2_O_3_ located in the upper layer will occur, as seen by Yuxiang Xu [32] after oxidizing AlCrN at 1100 °C for 20 h.

**Figure 11:**
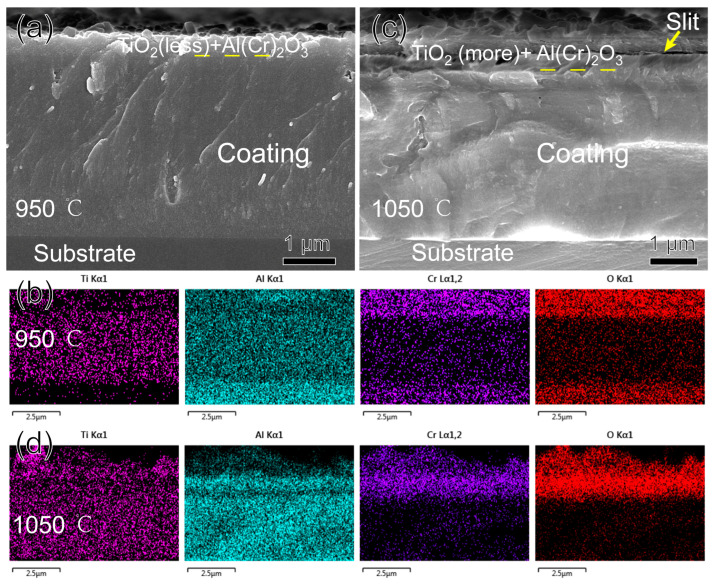
Morphology and EDS mapping of TiAlSiN/AlCrN coating fracture cross-section after oxidizing at high temperature for three hours: (**a**,**b**) 950 °C; (**c**,**d**) 1050 °C.

Figure 11 shows the morphology and EDS-mapping of the fracture cross-section of the TiAlSiN/AlCrN multilayer coating after oxidation for three hours at 950 °C and 1050 °C. At 950 °C, just as it is difficult to perceive the presence of the oxides in the XRD of Figure 8c, the thickness of the oxides shown in Figure 11a,b is significantly smaller than that of the other two monolayer coatings at this temperature. In addition, Al, Cr, or Ti oxides are not delaminated but mixed. This should be attributed to the numerous interlayer interfaces possessed by the multilayer coating, which act as barriers to oxygen and metal ion diffusion [33]. All metal ions, including Al, which has the strongest affinity for oxygen, are imprisoned in situ and oxidized. Therefore, the multilayer coating shows reduced oxidation and no delamination of the oxides at 950 °C. At 1050 °C, the oxide’s thickness increased, and the Ti concentration in it enhanced significantly, indicating that the TiO_2_ increased significantly. In addition, the same slit as the TiAlSiN coating appeared at the interface between the oxide and nitride, as shown in Figure 11c. All these phenomena are still caused by the r-TiO_2_ coming from the phase transition at this temperature. The r-TiO_2_ accompanying the pores weakens the multilayer structure’s hindering effect on diffusion, resulting in increased oxidation and a thicker oxide layer. In addition, the involvement of r-TiO_2_ makes it impossible to form a continuous dense (Cr, Al)_2_O_3_ film like AlCrN in the outermost layer of the oxide, so the surface of the oxide layer is rugged, as seen in Figure 11d. In this case, the barrier effect of the oxide layer decreases significantly. It has been proposed that Ti has a negative effect on the oxidation resistance of the AlCrN coating, attributed to the greater affinity of Ti for oxygen and the cracking of the (Cr, Al)₂O₃ surface film induced by the growth stress of TiO₂ [32]. However, in terms of affinity, Al has a greater affinity for O. In addition, the molar volume per unit oxygen atom of TiO_2_ lies between Al_2_O_3_ and Cr_2_O_3_, so TiO_2_ cannot be considered to have more significant growth stress. We argue that the more likely cause of TiO_2_ damage to the (Cr, Al)_2_O_3_ surface film is the thermal stress determined by its maximum coefficient of thermal expansion, rather than its growth stress.

### 3.4. High-Temperature Wear Resistance

**Figure 12:**
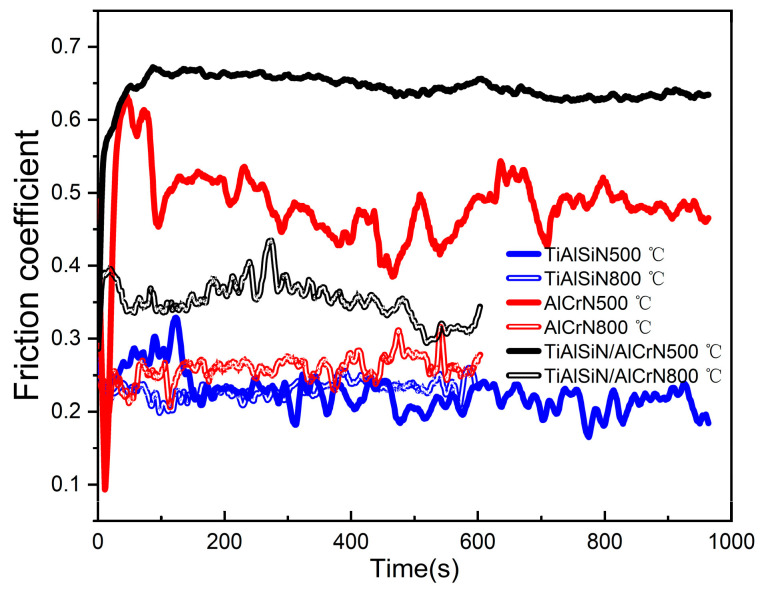
Friction coefficient curves of the three coatings at two temperatures.

**Figure 13:**
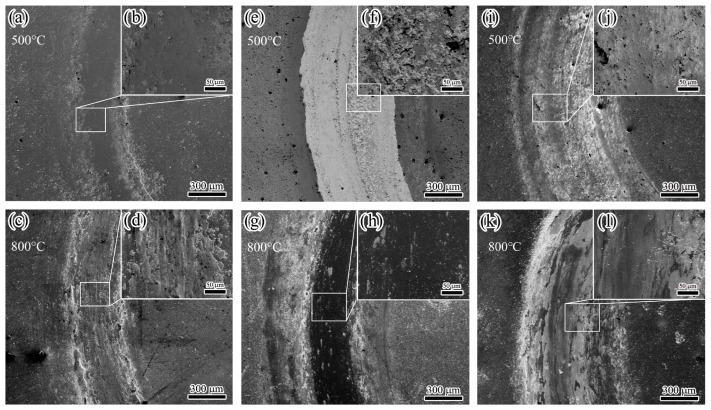
Wear tracks of three coatings after wear test at high temperatures: (**a**–**d**) TiAlSiN; (**e**–**h**) AlCrN; (**i**–**l**) TiAlSiN/AlCrN.

Figure 12 shows the friction coefficient curves of the three coatings, TiAlSiN, AlCrN, and TiAlSiN/AlCrN, after friction tests at two temperatures. First, the amplitudes of the curves at the two temperatures are analyzed. Compared to the ambient temperature of 800 °C, the curves of the three coatings at 500 °C fluctuate dramatically, especially for the AlCrN coating. This is caused by the original microscopic asperities and wear debris generated by friction on the contact surfaces. A large amount of wear debris is visible in SEM Figure 13f of the coating after the friction test. At 800 °C, the number of oxides involved in the friction process increases considerably. Although the XRD in Figure 8 does not show visible oxides, the friction surface temperature should be higher than the ambient temperature, so oxides exist. On one hand, these oxides fill in the grooves in the wear scar; on the other hand, they themselves act as a lubricant [8]. Second, the change in the mean friction coefficient from 500 °C to 800 °C is analyzed. The mean friction coefficient of the TiAlSiN/AlCrN coating decreases from 0.65 to 0.35 and that of the AlCrN coating decreases from 0.5 to 0.25, while that of the TiAlSiN coating shows no reduction. This is because the first two coatings both contain Cr₂O₃. Other researchers have also found that the lubricating effect of Cr₂O₃ reduces the friction coefficient [34]. The TiAlSiN coating achieves the lowest friction coefficient related to its smooth and hard surface. The TiAlSiN/AlCrN multilayer coating exhibits the highest friction coefficient, possibly due to the impact of the multilayer structure on the production of wear debris, requiring further investigation.

**Figure 14:**
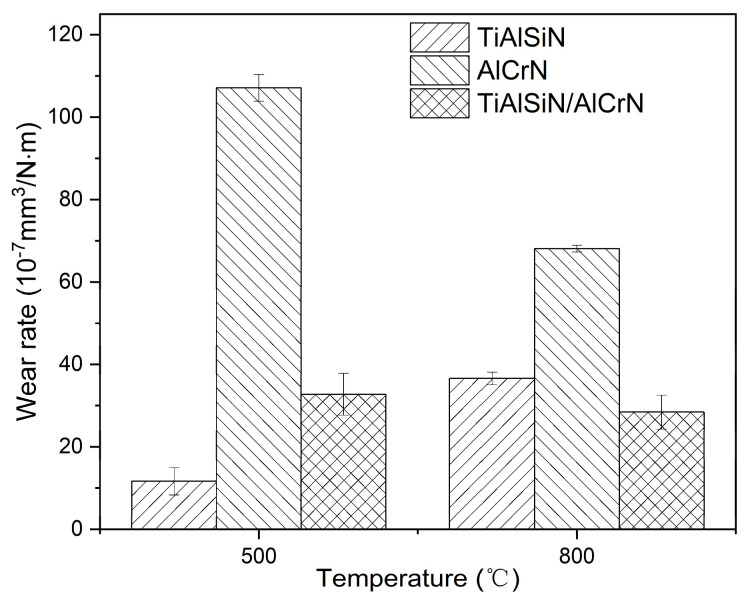
Wear rate of three coatings at high temperatures.

After the friction test, the morphology of the wear tracks of the three coatings is analyzed. At 500 °C, the AlCrN coating exhibited the most conspicuous wear tracks. The friction produced a large amount of debris, which accumulated on the wear tracks, as shown in Figure 13f. The TiAlSiN coating had imperceptible wear tracks; TiAlSiN/AlCrN was intermediate between the two. The wear rate histograms in Figure 14 support the above observations. The AlCrN coating has the highest wear rate, the TiAlSiN coating has the lowest, and the TiAlSiN/AlCrN coating is in the middle of the range. At 800 °C, compared to 500 °C, the wear tracks of the TiAlSiN coating became obvious, and a large amount of debris was generated, as shown in Figure 13d, indicating that the wear of this coating had worsened. In contrast, the wear tracks of the AlCrN and TiAlSiN/AlCrN coatings became smoother, suggesting that the wear of these two coatings was reduced. Again, the wear rate histograms support the above observation. The wear rate of the TiAlSiN coating increases with increasing temperature and exceeds that of TiAlSiN/AlCrN. In contrast, the wear rate of TiAlSiN/AlCrN and AlCrN does not increase but decreases. In addition, regardless of whether the temperature is 500 °C or 800 °C, the wear rate of AlCrN coating is the highest compared to the other two.

The explanation for the above results is firstly analyzed from the point of view of nanohardness and elastic modulus (Figure 4). The higher the H^3^/E*^2^ value, the stronger the resistance to plastic deformation and wear [22,23]. The order of the magnitude of the H^3^/E*^2^ values for each coating is TiAlSiN > TiAlSiN/AlCrN > AlCrN, which is consistent with the order of the resistance to wear of the coatings obtained from the friction tests at 500 °C. At 800 °C, although the temperature becomes higher, as AlCrN and TiAlSiN/AlCrN have higher onset oxidation temperatures than TiAlSiN, as shown in Figure 6, the H^3^/E*^2^ value still dominates the wear resistance of these two coatings, and then AlCrN still maintains the highest wear rate at 800 °C. However, for TiAlSiN, although the ambient temperature of 800 °C does not reach its onset of oxidation temperature of 900 °C, in the actual friction tests, the temperature of the friction surface should be higher than the ambient temperature, meaning that the TiAlSiN coating has been oxidized and is thus unstable. The result is that the wear rate of TiAlSiN overtakes that of TiAlSiN/AlCrN coating.

## 4. Conclusions

(1) TiAlSiN/AlCrN multilayer coating shows a mixed crystal structure of TiAlSiN and AlCrN in the phase; the nanohardness, elastic modulus, and adhesion strength of the multilayer coating conform to the “law of mixtures,” i.e., the above properties of TiAlSiN/AlCrN multilayer coating fall between those of the two monolayers. Benefiting from stress relief due to interfacial effects, TiAlSiN/AlCrN multilayer coating exhibits significantly lower residual stress than the two monolayer coatings.

(2) The order of oxidation resistance of the three coatings is TiAlSiN < TiAlSiN/AlCrN < AlCrN. The oxidation resistance of the TiAlSiN coating is attributed to the dense oxidation product Al_2_O_3_ and the nanocomposite structure formed by Si. However, the loose porous oxide of Ti, TiO_2_, negatively affects its oxidation resistance. The oxidation resistance of AlCrN coating benefits from the combined protection of the dense oxidation products Al_2_O_3_ and Cr_2_O_3_. The oxidation resistance of TiAlSiN/AlCrN multilayer coating inherits the advantages and disadvantages of the two monolayer coatings.

(3) The effect of the coatings’ mechanical properties and oxidation resistance on the wear resistance depends on the temperature. At 500 °C, the order of wear resistance of the three coatings is consistent with the order of H^3^/E*^2^ values, i.e., TiAlSiN > TiAlSiN/AlCrN > AlCrN; at 800 °C, the coating’s oxidation resistance plays a crucial role, so the order of wear resistance changes into TiAlSiN/AlCrN > TiAlSiN > AlCrN due to TiAlSiN oxidizing before TiAlSiN/AlCrN.

## Figures and Tables

**Table 1:** Coating thickness and composition measured by SEM and EDS.

Target	Coating	Thickness (μm)	Chemical Composition (at.%)				
			Al	Cr	Ti	Si	N
Ti_0.45_Al_0.45_Si_0.10_	TiAlSiN	3.69	20.22	−	22.66	3.89	53.23
Al_0.7_Cr_0.3_	AlCrN	3.63	33.81	18.14	−	−	48.05
Ti_0.45_Al_0.45_Si_0.10_ and Al_0.7_Cr_0.3_	TiAlSiN/AlCrN	3.52	27.43	7.38	11.71	2.27	51.21

## Data Availability

The original contributions presented in this study are included in the article. Further inquiries can be directed to the corresponding author.
